# Supplementary material for: Vital Conversations: An Interactive Conflict Resolution Training Session for Fourth-Year Medical Students
Source: MedEdPORTAL. 2021 Jan 25;17:11074. doi: 10.15766/mep_2374-8265.11074 (PMC7830754; doi:10.15766/mep_2374-8265.11074)
Supplement: Supplementary file 1 — Prework.docxTKI Teaching for Prework.docxVideo Realistic for Appendix A.mp4Video Empathic for Appendix A.mp4Rubric.docxClinical Encounter for Student.docxStandardized Patient Brief.docxPostwork.docxVideo 1 Conflict Resolution Postwork.mp4Video 2 Conflict Resolution Postwork.mp4 [file mep_2374-8265.11074-s001.zip › A. Prework.docx]

## APPENDIX A

## Conflict Resolution Session: Student Prework

Thank you for completing this pre-work for the conflict resolution exercise.

1. Please select your name from the following list. [*This question can be converted free-text.*]
2. Please select your coach and/or the faculty member who will be reviewing your video. [*This question can be converted free-text.*]

The next several questions will ask you demographic information (if you are willing to provide it) and your prior experience with conflict resolution.

1. How old are you? [*Free-text*]
2. With which gender do you identify?

- Male
- Female
- Neither
- I prefer not to answer

1. How would you describe your race?

- I identify myself as a member of an under-represented minority (African American, Hispanic or Latino, Pacific Islander, Native American)
- I identify myself as Caucasian, Asian, or Indian
- My race is not indicated and is: __________________________
- I prefer not to answer this question.

1. Have you ever taken a conflict management/resolution class?

- Yes
- No

1. How frequently do you use conflict resolution? (On a scale of 0-10, never to daily)
2. What does conflict resolution entail? i.e.—What does “conflict resolution” mean to you? Are there certain skills you use with conflict resolution?
3. Why is conflict resolution important in medicine?

Professional communication between individuals of the same profession (on your own team or on a consulting team) and between individuals of different professions (nurses and doctors) is a skill you will practice daily.  
 
Professional communication isn't easy.  We make mistakes all the time.  Some of these mistakes will waste time and money, and at its extreme, potentially even result in a poor patient outcome.  And sometimes, that communication will cause conflict.
 
Failures of professional communication are both so important and perhaps so frequent that they are represented in pop culture, both for humor and also for good drama.  

The following two short videos introduce the importance of and failures of professional communication.  Please watch them as an introduction to conflict resolution. **Of note, you do not have to use the videos listed above; it is OPTIONAL. To facilitate recognition of professional communication failures, provide video examples from any source.**

- Pop Culture Reference #1: <https://www.youtube.com/watch?v=5m3bpJ4hTG8>
- Pop Culture Reference #2: <https://www.youtube.com/watch?v=8gXCEc95cio>

In this next block, we will introduce ways in which individuals manage conflict resolution, and we will ask you to reflect on the method you most often use.  Please refer to Appendix B for this material.

Appendix B’s descriptions of the modes are to help introduce you to the subject of conflict resolution. It is not supposed to take the place of conflict management courses. Our goal is to show you that each mode has advantages and disadvantages. Knowing when and how to effectively use each mode is key to successful conflict resolution.

1. Based upon what you have read, what mode do you think you are and why?

In this next section, you will be asked to critically evaluate videos of conflict in the medical setting. You will be evaluated on these same behaviors and actions during your stimulated encounter.

*Of note, the number of videos and analysis can be changed by the curriculum designers. There are 2 required ones shown.*

This video demonstrates a resident-attending physician encounter. (Video: <https://www.youtube.com/watch?v=CyrSuY5g8iA>; this video is provided in Appendix C)

1. When did the conflict start? Who started the conflict? [*Free-text*]
2. List the moments of positive and negative body language. [*Free-text*]
3. Describe any moments of hostility, conflict avoidance, and/or accommodation. Include WHO was hostile/avoiding/accommodating. [*Free-text*]
4. Describe moments of ineffective and effective listening. Could the conflict have gone differently if effective listening had taken place? Were there moments when concerns were ignored or validated? [*Free-text*]
5. Were there any open-ended questions used or were they all close-ended? [*Free-text*]
6. Was the conflict resolved? Who resolved the conflict? [*Free-text*]
7. How much of the conversation was driven by emotion?

(On a scale of 0-10, none to the entire conversation)

1. How patient-centered was the conversation?

(On a scale of 0-10, none to the entire conversation)

1. How would you have handled the conversation differently? [*Free-text*]
2. Have you ever witnessed an encounter like this? If so, please describe it briefly below. [*Free-text*]

This next video demonstrates the same resident-attending physician encounter, but in a different way. (Video: <https://www.youtube.com/watch?v=VHhqSIbY51s>; this video is provided in Appendix D)

1. What was done differently in this version than in the previous? [*Free-text*]

Please watch the following video. We will again ask you to comment on several specific parts of the video for aspects of conflict resolution. (**Provide a video of your choice here**; an example, OPTIONAL video is shown at the following link--<https://www.youtube.com/watch?v=YJ2eEz5-7oE>)

1. When did the conflict start? Who started the conflict? [*Free-text*]
2. List the moments of positive and negative body language. [*Free-text*]
3. Describe any moments of hostility, conflict avoidance, and/or accommodation. Include WHO was hostile/avoiding/accommodating. [*Free-text*]
4. Describe moments of ineffective and effective listening. Could the conflict have gone differently if effective listening had taken place? Were there moments when concerns were ignored or validated? [*Free-text*]
5. Were there any open-ended questions used or were they all close-ended? [*Free-text*]
6. Was the conflict resolved? Who resolved the conflict? [*Free-text*]
7. How much of the conversation was driven by emotion?

(On a scale of 0-10, none to the entire conversation)

1. How patient-centered was the conversation?

(On a scale of 0-10, none to the entire conversation)

1. How would you have handled the conversation differently? [*Free-text*]
2. Have you ever witnessed an encounter like this? If so, please describe it briefly below. [*Free-text*]

You have now completed the pre-work assignment. The following questions ask you for feedback on this assignment.

1. This assignment was (select all that apply):

- Resulted in new learning
- Was a good review
- Prompted self-reflection
- Will change the way I practice
- Makes me want to investigate more about this topic
- Changed my perceptions on this topic
- None of the above

1. How much time did it take you to complete this exercise?

- 15-30 minutes
- 30-60 minutes
- 60-90 minutes
- >90 minutes

1. Next year:

- This pre-work session should be repeated (in current form)
- This pre-work session should be modified (see question #38)
- This pre-work session should be mandatory
- This assignment should not be included in Capstone

1. How should we modify it? [*Free-text*]

**Videos Included:**

Included as Appendix C

Video by [PhysicianHealthBC], retrieved from: [https://www.youtube.com/watch?v=CyrSuY5g8iA] on [29 FEB 2020]. Attribution Creative Commons License associated: [CC https://www.youtube.com/watch?v=CyrSuY5g8iA]

Included as Appendix D

Video by [PhysicianHealthBC], retrieved from: [https://www.youtube.com/watch?v=VHhqSIbY51s] on [29 FEB 2020]. Attribution Creative Commons License associated: [CC https://www.youtube.com/watch?v=VHhqSIbY51s]
