# Supplementary material for: Vital Conversations: An Interactive Conflict Resolution Training Session for Fourth-Year Medical Students
Source: MedEdPORTAL. 2021 Jan 25;17:11074. doi: 10.15766/mep_2374-8265.11074 (PMC7830754; doi:10.15766/mep_2374-8265.11074)
Supplement: Supplementary file 1 — Prework.docxTKI Teaching for Prework.docxVideo Realistic for Appendix A.mp4Video Empathic for Appendix A.mp4Rubric.docxClinical Encounter for Student.docxStandardized Patient Brief.docxPostwork.docxVideo 1 Conflict Resolution Postwork.mp4Video 2 Conflict Resolution Postwork.mp4 [file mep_2374-8265.11074-s001.zip › B. TKI Teaching for Prework.docx]

## APPENDIX B

## Thomas-Kilmann Conflict Modes

The Thomas-Kilmann Conflict Mode Instrument, also known as TKI, is the most commonly used tool to assess how a person behaves in a conflict.

The instrument looks at two components of a person’s behavior in order to determine their preferred mode: (1) assertiveness and (2) cooperativeness. Assertiveness describes the extent to satisfy one’s own concerns. Cooperativeness describes the extent to satisfy other’s concerns. Based upon these two axes, FIVE conflict resolution styles are defined. This can graphically be seen in Figure 1.


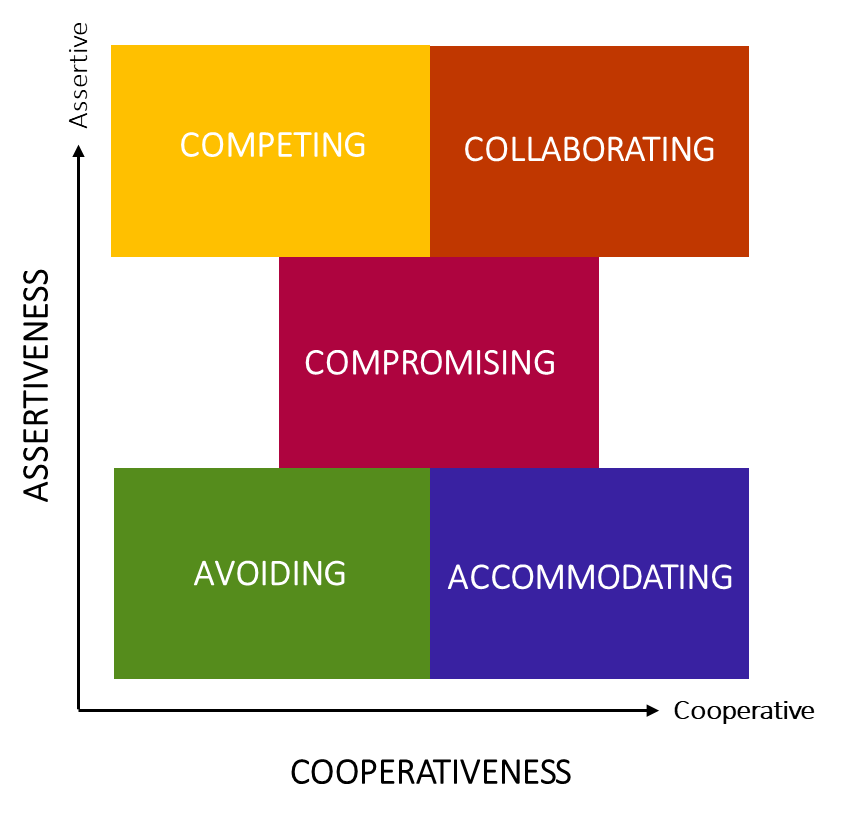


Figure 1: Conflict styles with varying amounts of cooperativeness and assertiveness. Adapted from the Thomas Kilmann Instrument (TKI), this image is owned by the authors.

Now, we will go through each of the conflict resolution modes. The following is adapted from the TKI Report. We will describe the mode itself, when the mode is used, contributions of that mode to a team, and questions to ask yourself if it is being underused or overused. We have also included situations in medicine where it may be the preferred mode. It is important to remember that there is not ONE correct mode. Everyone is capable of using all five modes; however, most use a particular mode more, develop skills in that mode, and subsequently, use that mode more in conflicts, even if it is not the most appropriate.

At the end, we will ask you which type of conflict resolution you believe you use most often. It is a result of both your personal predispositions and requirements of the conflict you are in.

**COLLABORATING**: On one extreme of the axis, this style is both very assertive and very cooperative. A collaborator attempts to work with the other person to find a solution that fully satisfies the concerns of both parties for a “win/win”.

Collaborators see conflicts as problems to be solved and want quality decisions that resolve the issue. They believe in the power of consensus and in sharing information and understandings. Teammates are seen as allies and those on the outside are seen as potential allies. They build on other’s ideas and listen well. Collaborators value innovation, open-mindedness, learning, and consensus.

In a team, collaborators are helpful in reaching win-win solutions that provide long-term resolution to a conflict issue. They find superior, high-quality solutions to important issues and can be sources of creativity and innovation.

Situations to use this mode:

- An integrative solution is needed and concerns of both parties are too important to be compromised
- The objective is to learn, test one’s assumptions, and understand others’ views
- Merge insights from people with different perspectives on a problem
- Gain commitment by incorporating others’ concerns into a consensual decision
- Work through hard feels that have been interfering with a relationship

Signs of overuse

- Do you spend time discussing issues in depth that don’t seem to warrant it?
  - Collaboration takes time and energy. Small problems don’t require optimal solutions, and not all personal differences need to be hashed out. The overuse of this style sometimes represents the desire to minimize risk by diffusing the responsibility for a decision or by postponing an action.
- Does your collaborative behavior fail to elicit collaborative responses from others?
  - The nature of collaborative behavior may make it easy for others to disregard proposals or take advantage of one’s trust. Cues that would indicate defensiveness, strong feelings, impatience, competitiveness, or conflicting interests may be missed.

Signs of underuse

- Is it difficult to see differences as opportunities for joint gain, learning, or problem solving?
  - Approaching all conflicts with pessimism can prevent people from seeing collaborative possibilities.
- Are other uncommitted to your decisions or policies?
  - Their concerns may not be incorporated into those decisions or policies.

In medicine, consider these situations in which a collaborative style can be used:

- A multidisciplinary family meeting
- Discharge plans for a patient
- Hospital quality improvements

**AVOIDING**: On the opposite extreme from collaborating, this style is both unassertive and uncooperative. An avoider does not immediately pursue his or her own concerns or those of the other person; overall, he or she does not address conflict.

Avoiders see conflicts as intrusions or disruptions that can divert energy from work and cause unnecessary stress. They believe in using time wisely, addressing only important issues when the conditions are right. Teammates are often regarded as potential time sinks, so avoiders like to keep meetings short and often defer them until they have more time. Avoiders value time, energy, and being prepared.

In a team, avoiders are sensitive to time demands and stress of conflict issues. They also promote prudence when dangerous issues, such as highly sensitive topics or political issues with hidden agendas, arise, and help postpose issue until conditions improve to address them.

Situations to use this mode:

- An issue is unimportant or when another issue is more important
- There is no change of satisfying your concerns, especially when the other has more power or when the issue is something that would be very difficult to change
- Potential costs of confronting a conflict outweigh the benefits of its resolution
- When people need to cool down, reduce tensions to a productive level and to regain perspective and composure
- Gathering more information outweighs advantages of immediate decision
- Others can resolve the issue more effectively
- Issue is due to another basic issue

Signs of overuse

- Does coordination suffer because people sometimes have trouble getting your input on issues?
- Does it sometimes appear that people are “walking on eggshells”?
  - There is too much energy devoted to caution and avoiding issues, indicating those issues need to be faced and resolved.
- Are decisions on important issues sometimes made by default?
  - No engagement in the discussion means one’s concerns are overlooked

Signs of underuse

- Are people’s feelings hurt?
  - Discretion and tact should be used. Issues can be framed in other, nonthreatening ways.
- Do you feel rushed or overwhelmed by multiple issues?
  - Set priorities. Less important issues can be avoided or delegated to others.

In medicine, consider these situations in which an avoiding style can be used:

- Political arguments among attending physicians
- Delegating an intern to answer floor nursing concerns while you admit someone to the ICU
- Personal disagreements of co-residents

**ACCOMMODATING**: This style is unassertive but cooperative. Accommodators neglect their own concerns to satisfy concerns of others.

Accommodators see conflicts as social/emotional issues to be settled with support and sensitivity. They regard teammates as friends and value support, generosity, goodwill, and team cohesiveness. They see compassion and friendship as more important in conflicts than the smaller issues involved.

In a team, accommodators’ compassion and generosity can serve an important role in interpersonal relations. They help maintain goodwill and trust along with psychological support. They serve as peacekeepers to restore harmony and soothe hurt feelings.

Situations to use this mode:

- When you realize you are wrong
- The issue is much more important to the other party than it is to you
- Build up social credits for later issues more important to you
- Competing would only damage your cause
- Preserving harmony and avoiding disruption are important
- Help others experiment and learn from their mistakes

Signs of overuse

- Do you feel that your ideas and concerns sometimes don’t get the attention they deserve?
  - Deferring too much can deprive you of influence, respect, and recognition.
- Is discipline lax?
  - Some rules, procedures, and assignments are important and need to be enforced. Being too accommodating may harm you, others, or the organization.

Signs of underuse

- Having trouble building goodwill
- Being viewed as unreasonable or failing to recognize legitimate exceptions to the rules
- Having trouble admitting one is wrong
- Refusing to give up

In medicine, consider these situations in which an accommodating style can be used:

- Teaching new interns or medical students on your team
- Helping a colleague with his or her tasks if he or she is overwhelmed with a sick patient
- Allowing family members to guide the conversation about their concerns for discharge

**COMPETING**: This style is assertive, but not cooperative; this is a power-oriented style. Competitors pursue their own concerns at other’s expense, using whatever power seems appropriate to win.

Competitors see conflicts as contests that they need to win. They regard teammates with other views as opponents and will take on the entire group if an issue is important to them. They value tough-mindedness, candor, courage, and effective implementation.

In a team, competitors are powerful advocates for positions they believe are valid. They can face touch facts and push for decisions that may be unpopular. They can be an effective voice compared to others who are too polite to take action.

Situations to use this mode:

- Quick, decisive action is needed
- On issues where unpopular courses of action need implementing
- Issues that are vital to welfare when you know you’re right
- Protect oneself from people who try to take advantage of noncompetitive behavior

Signs of overuse

- Are you surrounded by “yes people”?
  - Other have learned that it is unwise to disagree with you. This can close you off from information.
- Are others afraid to admit ignorance and uncertainties to you?
  - People are less likely to ask for information or opinions because of how certain and confident you are

Signs of underuse

- Feeling powerless in situations
  - You may be unaware of the power you have or may be uncomfortable with the idea of using it, hindering your effectiveness.
- Having trouble taking a firm stand, even when one sees the need
  - Concerns for others’ feelings or anxieties and postponing decisions may add to the suffering or resentment or others.

In medicine, consider these situations in which a competing style can be used:

- Leader in a Code Blue
- Surgeon in the operating room for a patient that is profusely bleeding
- Meetings where decisions regarding call need to be made

**COMPROMISING**: This is in the middle for both assertiveness and cooperativeness. Compromisers find expedient, mutually acceptable solutions that **partially** satisfies both parties. Otherwise known as the “Middle Ground”.

Compromisers see conflict as a chance to find the middle ground and make deals. They are flexible and are reasonable, avoiding extreme positions. They regard teammates as amicable negotiating partners and value moderation, reasonableness, and pragmatism. They seek favorable settlements using soft bargaining to achieve fairness.

In a team, compromisers provide a practical force for moderation. When collaborators are unable to find a win-win, compromisers suggest temporary solutions. They put less strain on goodwill than competitive styles and take less time than collaborative styles.

Situations to use this mode:

- Goals are moderately important but not worth the effort or potential disruption of more assertive modes
- Both sides with equal power are strongly committed to mutually exclusive goals
- Achieve a temporary settlement of a complex issue
- Arrive at an good solution under time pressure
- Backup when collaboration or competition fails

Signs of overuse

- Do you concentrate so heavily on the practicalities and tactics of compromise that you lose sight of the larger issue?
  - This can lead to unintended and costly compromises of principles, values, and long-term objectives.
- Does an emphasis on bargaining and trading create a cynical climate of gamesmanship?
  - This may undermine interpersonal trust and deflect attention from the issue being discussed.

Signs of underuse

- Being too sensitive or embarrassed to engage in the give-and-take of bargaining
  - This prevents the other party from getting a fair share in negotiations.
- Finding it difficult to make concessions
  - You may have trouble getting out of a mutually destructive argument or power struggle.

In medicine, consider these situations in which a collaborating style can be used:

- Switching call with co-residents
- Working with nurses on care orders to ensure patient care is not compromised and the nurses are not being overtasked
- Working on research projects and dividing up lab responsibilities
