# Supplementary material for: Vital Conversations: An Interactive Conflict Resolution Training Session for Fourth-Year Medical Students
Source: MedEdPORTAL. 2021 Jan 25;17:11074. doi: 10.15766/mep_2374-8265.11074 (PMC7830754; doi:10.15766/mep_2374-8265.11074)
Supplement: Supplementary file 1 — Prework.docxTKI Teaching for Prework.docxVideo Realistic for Appendix A.mp4Video Empathic for Appendix A.mp4Rubric.docxClinical Encounter for Student.docxStandardized Patient Brief.docxPostwork.docxVideo 1 Conflict Resolution Postwork.mp4Video 2 Conflict Resolution Postwork.mp4 [file mep_2374-8265.11074-s001.zip › E. Rubric.docx]

## Appendix E: Rubric

After this encounter, you, the standardized actor, and your coach will evaluate your video for the following:

- Effective listening,
- Validating others concerns,
- Using open ended questions,
- Body stature and tone,
- Ability to control emotions, and
- Remaining Patient Centered.

Conflict management will also be assessed:

- Did you acknowledge there was a problem?
- Did you break the problem down into smaller issues?
- Did you acknowledge shared decision making?, and
- Did you summarize commit to a collaborative plan?

At the end of the assignment, you will be assessed for entrustment for Core EPA #9 Interprofessional Teamwork.

The Liaison Committee on Medical Education (LCME) defines entrustment as trustworthiness in applying knowledge, skills, and attitudes in performance. Being trustworthy is knowing the limits of your knowledge, seeking help, and truthfulness.

Behaviors that demonstrate Entrustment in EPA #9:

- Acts as an active and integrated member of the team who in most situations prioritizes team goals over one’s own professional goals.
- Understands the roles of other team members, seeks their counsel, actively listens to their recommendations and incorporates them into practice.
- Typically communicates in a bidirectional manner and keeps all team members informed and up to date.
- Modifies and adapts communication content and style based on audience, venue, receiver preference or type of message.
- In most situations is able to read one’s own emotions and anticipates and reads the emotions of others.
- Maintains a professional demeanor in all but the most trying of circumstances.
- Actively engages with the patient and other team members fosters the ability to coordinate care and
- provide for seamless transitions between care providers/settings

Pre-entrustment means that you still need development in skills that would make you entrustable to do tasks independently as an intern.

Behaviors that demonstrate Pre-Entrustment:

- Prioritizes one’s own goals over those of the team due to performance anxiety.
- Demonstrates limited understanding of the roles of other team members besides physicians, (e.g. seeks counsel from the other physicians to the exclusion of other team members).
- Typically communicates in a unidirectional manner and in response to a prompt.
- Displays limited ability to modify communication based on audience, venue, receiver preference or type of message.
- Demonstrates difficulty reading his own emotions and struggles to anticipate or read the emotions of others.
- Succumbs to lapses in professionalism particularly when stressed or tired.
- Is typically a more passive member of the team and has limited interaction with other team members with the unintended consequence of not being able to optimally support patients through transitions of care

Below, you will find the questions for reference that you, your faculty coach, and the SP will use to provide feedback. These questions will be in your post-work for you to self-critique yourself.

1. During this encounter, please note how long it took from the start of encounter until:

|  | Time (seconds) |
| --- | --- |
| The student to realize there was a conflict |  |
| For the two individuals to ask each other what their concerns/interests were |  |
| For the two individuals to find a compromise |  |
| Total duration of the encounter |  |

1. Rate your ability to negotiate:

|  | Not at all | A little bit | A moderate amount | Mostly/Completely |
| --- | --- | --- | --- | --- |
| Identify/acknowledge there were different priorities/a problem |  |  |  |  |
| Break the problem down into smaller pieces |  |  |  |  |
| Acknowledge a shared goal |  |  |  |  |
| Summarize a compromise/commit to a plan that works for both parties |  |  |  |  |

1. Rate your ability to listen: [Scale from 0 to 10]
   1. 0 (Ineffective listening, interrupts, explains too much)
   2. 10 (active listening, no interruptions)
2. Rate your ability to acknowledge your concerns : [Scale from 0 to 10]
   1. 0 (ignores concerns of others, dismisses, minimizes)
   2. 10 (validates concerns, restates them, summarizes them)
3. Rate your ability to ask questions effectively: [Scale from 0 to 10]
   1. 0 (uses all closed ended questions)
   2. 10 (uses all open ended questions)
4. Rate your ability to use appropriate body language: [Scale from 0 to 10]
   1. 0 (uses body language that shuts others out, crosses arms, enters others physical space)
   2. 10 (uses body statue, tone, and eye contact that encourages discussion)
5. Rate your emotional intelligence: [Scale from 0 to 10]
   1. 0 (emotions drive conversation, frustration, anger, lack of empathy)
   2. 10 (able to control emotions during encounter, uses empathy, understanding)]
6. Rate your overall approach to the conversation: [Scale from 0 to 10]
   1. 0 (made situation personal, needs to win)
   2. 10 (avoids making situation personal, remain patient-centered)
7. What are some ways you could improve your approach to this conversation? [*Free-text*]
