# Supplementary material for: Vital Conversations: An Interactive Conflict Resolution Training Session for Fourth-Year Medical Students
Source: MedEdPORTAL. 2021 Jan 25;17:11074. doi: 10.15766/mep_2374-8265.11074 (PMC7830754; doi:10.15766/mep_2374-8265.11074)
Supplement: Supplementary file 1 — Prework.docxTKI Teaching for Prework.docxVideo Realistic for Appendix A.mp4Video Empathic for Appendix A.mp4Rubric.docxClinical Encounter for Student.docxStandardized Patient Brief.docxPostwork.docxVideo 1 Conflict Resolution Postwork.mp4Video 2 Conflict Resolution Postwork.mp4 [file mep_2374-8265.11074-s001.zip › F. Clinical Encounter for Student.docx]

## APPENDIX F

## Conflict Resolution Session: Clinical Encounter, Student Brief

You will come to the clinical skills center for a standardized patient encounter. The standardized patients is a nurse. During the encounter, a conflict will arise, and you will be asked to manage that conflict. We will provide the clinical scenario here, but it will also be provided to you at the time you meet arrive to the clinical skills center.

**Clinical Scenario:** You have had a busy day on clinical service. You are just getting ready to leave for the day. You have found it useful this year as an intern to check in with the nurses before you leave to make sure that all the plans set during rounds have unfolded as expected. You run into Nurse Thompson, the nurse managing some of your patients. **“Hi, I just wanted to see if you had any updates or questions about patients on our team.”**

The nurse will ask you about Mr. Howard.

**The Patient:** Mr. Howard is a 45 year old male, type 1 diabetic admitted with diabetic ketoacidosis (DKA) and sepsis secondary to an infected diabetic foot ulcer. He also has chronic kidney disease (CKD) stage 4, hypertension (HTN), prior stroke, and prior upper extremity deep vein thrombosis (DVT). Mr. Howard has been on antibiotics for 12 hours and his condition is stable, though still serious. He has received 3 liters of intravenous (IV) fluid boluses and his vitals are now better: Temperature 37.8 degrees Celsius; heart rate 105; blood pressure 100/54; respiratory rate 18; and 98% oxygen saturation on room air.

**Past Medical History:**

1. Stage 4 CKD, with baseline Cr of 2.8
2. Prior stroke
3. Prior upper extremity DVT—related to previous line
4. Diabetes Mellitus, type 1 since age of 7, multiple admissions for DKA –diabetes is quite brittle. Patient hypoglycemic unaware. Complications include retinopathy, neuropathy, and autonomic instability.
5. Hyperlipidemia (HLD)
6. Hypertension (HTN), also now has hypotension with autonomic instability
7. Erectile Dysfunction

**Current Medications**

- Insulin drip running at 10U/hour
- D5NS with 20KCl running at 125cc/hour
- Vancomycin 1g and following vancomycin levels
- Piperacillin/Tazobactam (Zosyn) 2.25g IV Q6Hr
- Heparin 5000U SC BID
- Atorvastatin 40mg PO QD
- Aspirin 325mg PO QD

**Plan:**

1. DKA: Improving; the patient’s last glucose was 185 and the gap is closing (currently 16); you’ve added D5 and potassium to the fluids with a plan to transition to subcutaneous (SC) insulin soon.
2. Diabetic foot ulcer: the team is concerned about possible osteomyelitis and will be getting a MRI today to evaluate. You are consulting orthopedics and wound management today. Currently Day 2 of vancomycin and zosyn, both renally adjusted.
3. Hypotension: resolving. The patient required 3 liters of fluid, but his blood pressure has improved. Blood cultures are pending. Patient also has autonomic dysfunction and sometimes has low blood pressures at home. Recently, they have stopped has anti-hypertensives, and you suspect his BP is low for this reason. Endocrinology to weigh on managing the autonomic instability.
4. CKD: the patient’s creatinine was up to 3.8 when he arrived; it is already going back down. Nephrology is aware. No PICCs to preserve future use of veins for arteriovenous (AV) fistula.
5. Stroke/HLD: currently on home doses of statins and aspirin
6. Electrolytes: Potassium (K) was last 3.8, added KCl to his fluids. Currently NPO (nothing by mouth), but once gap closes and SC insulin started, you will change his diet. Won’t need fluids much longer
7. Prophylaxis: on SC heparin, adjusted for renal dose. No need for stress ulcer prophylaxis; be on alert for DVT since patient has had one in the past.
8. IV access: Trying to avoid a central line due to risk of infection, need to preserve sites for future AV fistula, and nephrology’s request to not place a PICC. IV team has been able to get 2 peripheral IVs. Patient has complained about getting stuck multiple times getting labs and has required some encouragement from the team in order to avoid a central line for the reasons stated.

This plan was developed by you and your senior resident and approved by the attending physician.

Once you walk into the room, you will encounter the patient’s nurse and will begin the simulation.
