# Supplementary material for: Vital Conversations: An Interactive Conflict Resolution Training Session for Fourth-Year Medical Students
Source: MedEdPORTAL. 2021 Jan 25;17:11074. doi: 10.15766/mep_2374-8265.11074 (PMC7830754; doi:10.15766/mep_2374-8265.11074)
Supplement: Supplementary file 1 — Prework.docxTKI Teaching for Prework.docxVideo Realistic for Appendix A.mp4Video Empathic for Appendix A.mp4Rubric.docxClinical Encounter for Student.docxStandardized Patient Brief.docxPostwork.docxVideo 1 Conflict Resolution Postwork.mp4Video 2 Conflict Resolution Postwork.mp4 [file mep_2374-8265.11074-s001.zip › G. Standardized Patient Brief.docx]

## APPENDIX G: Standardized Patient Brief

Conflict Resolution Session: Clinical Encounter, Standardized Patient Brief

Primary Case Author: Dr. Alison Clay

Secondary Case Author: Dr. Nancy Knudsen

Standardized Patient Educator: Kelly Bradford, Dan Sipp, Course Faculty

Name of Case: Vascular Access

Name of educational and or assessment activity: Conflict Resolution Session

Patient Name: Mr. Howard

Chief Complaint: Altered mental status

Most likely Diagnosis and Differential with rationale from history and/or physical exam:

Admitted for treatment of DKA and sepsis secondary to an infected diabetic foot ulcer

Challenge question to intern on medical team (student):

“Can we talk about Mr. Howard’s plan for the day?”

Domains: Check all that apply

X Professionalism

X Communication and Interpersonal skills

- Medical History
- Physical exam
- Shared Decision Making
- Patient Education
- Clinical Reasoning
- Documentation
- Handoff
- Presentation
- Other:

Type and level of learner: MS3 or MS4

Case Objectives: please list specific objectives for each of the domains you have checked above:

1. To practice resolving a conflict in a professional manner
2. To recognize the direct and indirect behaviors that result in successful resolution of a conflict

| SETTING: outpatient, in patient, ED, home, nursing home, rehab, group etc. | Inpatient |
| --- | --- |
| SP PROFILE: This is the information about the standardized patient who will act as a nurse in this scenario. | |
| Age range | 30-50s |
| Sex (e.g., male, female, intersex, transwoman, transman) | Female (Can be male) |
| Race/ethnicity: | Any |
| Physical description (e.g., BMI, height range) | Any |
| Affect (e.g., pleasant, cooperative) | Assertive, busy finishing things towards the end of the shift |
| Education | Inpatient nurse |
| Level of health literacy | High |
| Typical day - what is the usual daily routine | Busy inpatient unit; towards shift change |

| CASE INFORMATION | |
| --- | --- |
| Chief Concern: Background and what the RN will say when greeted by the student | You, as the RN taking care of Mr. Howard, have been having trouble maintaining IV access. You and other nurses spend a lot of time obtaining access once it is lost. You and your nursing team believe that he needs more definitive access (midline, PICC, central line, etc.) and don’t understand the reasons the medical team is avoiding it.  You know that the intern (student) comes around nursing shift change to ask if there are any concerns or updates on patients for the day. Once the intern asks you about updates/plans, you will ask: “Can we discuss Mr. Howard’s plan of the day?” |
| Case and Additional Concerns: Other, if any, concerns the patient has today (i.e., symptoms, requests, expectations, etc.) that will become part of set agenda. | You and the other nurses are having trouble maintaining his IV access, and the patient is complaining about the many sticks he has been getting for labs, sometimes refusing labs until the nurses speak with him about the importance. The medical team seems to be happy with the current IV access.  There is a concern for osteomyelitis, so you believe that he will need some type of long-term access, even though you have heard Nephrology would like to avoid the PICC. |
| Nurse’s perspective and behavior: | After your question, the intern should provide you with updates on the patient. Once he or she is done, if not stated in the plan, you should ask specifically about vascular access. The student should explain the reasons why the team wants to maintain peripheral vascular access. If the student does not provide reasons, you should ask questions or be skeptical that it is a good idea to maintain access. You should stick to “the patient needs better access” until the student asks you why you are concerned about the access. The student does not know that one IV has been lost and only one remains; this happened after the team rounded, and you were just about to call the team when the student shows up to wrap up the day.  Your behavior should be that of someone who is busy and distracted because you are trying to get things together for the nurse coming on shift. Sit at the computer in the room, and type on the computer as if you are entering orders, or looking up patient values. Divert your gaze to the computer screen. Talk quickly, as if you are in a rush. The student should be able to recognize you are busy and ask you to pause to discuss the patient.  The student should recognize that you are not happy with the current access, and he or she should ask you WHY we cannot get by with one access (answers include incompatible medications and medications scheduled at the same time of day—IV insulin, antibiotics, etc).  The student should listen actively and ask you any clarifying questions prior to trying to come to a solution. While you should be pushy for a solution earlier on, once the student tries to come up with a solution, work with him or her, and understand the reasons the medical team wants to avoid central access.  If the student tries to partner with you, you can offer solutions to them such as “we could transition to subcutaneous insulin”, “we could ask the pharmacist to stagger the medications”, and “sometimes we place an external jugular IV, but this must be placed by the providers on the team”.  The case should end once you and the student have come to a point at which the student has explained the reasons the medical team wants to strongly avoid central access AND that the patient is getting better from DKA so should have less intravenous medications soon. The student should also explain that the status of the patient’s osteomyelitis is still unknown since an MRI is still pending and orthopedics hasn’t evaluated the patient yet. The student should express, in some way, that he or she understands your concerns as well as the patient’s discomfort with the sticks. It is okay to end the case if the student states that they can evaluate the need of labs or intravenous medications every day or speak to the patient about the important of avoiding central access due his potential need for dialysis in the future. It is also okay if the student states that they have to run any plan through the medical team for final approval. |
| THE PATIENT STORY: | Mr. Howard is a 45 year old male, type 1 diabetic admitted with diabetic ketoacidosis (DKA) and sepsis secondary to an infected diabetic foot ulcer. He also has chronic kidney disease (CKD) stage 4, hypertension (HTN), prior stroke, and prior upper extremity deep vein thrombosis (DVT). Mr. Howard has been on antibiotics for 12 hours and his condition is stable, though still serious. He has received 3 liters of intravenous (IV) fluid boluses and his vitals are now better: Temperature 37.8 degrees Celsius; heart rate 105; blood pressure 100/54; respiratory rate 18; and 98% oxygen saturation on room air.  **Past Medical History:**   1. Stage 4 CKD, with baseline Cr of 2.8 2. Prior stroke 3. Prior upper extremity DVT—related to previous line 4. Diabetes Mellitus, type 1 since age of 7, multiple admissions for DKA –diabetes is quite brittle. Patient hypoglycemic unaware. Complications include retinopathy, neuropathy, and autonomic instability. 5. Hyperlipidemia (HLD) 6. Hypertension (HTN), also now has hypotension with autonomic instability 7. Erectile Dysfunction   **Current Medications**   - Insulin drip running at 10U/hour - D5NS with 20KCl running at 125cc/hour - Vancomycin 1g and following vancomycin levels - Piperacillin/Tazobactam (Zosyn) 2.25g IV Q6Hr - Heparin 5000U SC BID - Atorvastatin 40mg PO QD - Aspirin 325mg PO QD   **Plan:**   1. DKA: Improving; the patient’s last glucose was 185 and the gap is closing (currently 16); the medical team has transitioned to D5 and potassium to the fluids with a plan to transition to subcutaneous (SC) insulin soon. 2. Diabetic foot ulcer: the medical team is concerned about possible osteomyelitis and will be getting a MRI today to evaluate. Currently Day 2 of vancomycin and zosyn, both renally adjusted. 3. Hypotension: resolving. The patient required 3 liters of fluid, but his blood pressure has improved. Blood cultures are pending. Patient also has autonomic dysfunction and sometimes has low blood pressures at home. The team thinks his BP is low for this reason. Endocrinology has been following the autonomic instability. 4. CKD: the patient’s creatinine was up to 3.8 on admission; it is already going back down. Nephrology is aware. 5. Stroke/HLD: currently on home doses of statins and aspirin 6. Electrolytes: Potassium (K) was last 3.8, added KCl to his fluids. Currently NPO (nothing by mouth), but once gap closes and SC insulin started, the medical team will change his diet. 7. Prophylaxis: on SC heparin, adjusted for renal dose. No need for stress ulcer prophylaxis; be on alert for DVT since patient has had one in the past. 8. IV access: IV team has been able to get 2 peripheral IVs. The IVs have failed in the past and it takes time for nursing to obtain new access. Patient has complained about getting stuck multiple times for labs and has required some encouragement from nursing. The medical team seems happy with the peripheral IVs and has encouraged the patient to avoid central lines. |
| PROFESSIONALISM ISSUES OR CHALLENGES: | At the end of this encounter, please provide the student with feedback using the following rubric. |

Rubric to Use to Evaluate the Student:

1. During this encounter, please note how long it took from the start of encounter for:
   1. The student to realize there is a conflict
   2. The student to ask what your concerns/interests were
   3. To find a compromise
2. Rate the student’s ability to negotiate:

|  | Not at all | A little bit | A moderate amount | Mostly/Completely |
| --- | --- | --- | --- | --- |
| Identify/acknowledge there were different priorities/a problem |  |  |  |  |
| Break the problem down into smaller pieces |  |  |  |  |
| Acknowledge a shared goal |  |  |  |  |
| Summarize a compromise/commit to a plan that works for both parties |  |  |  |  |

1. Rate the student’s ability to listen: [Scale from 0 to 10]
   1. 0 (Ineffective listening, interrupts, explains too much)
   2. 10 (active listening, no interruptions)
2. Rate the student’s ability to acknowledge your concerns : [Scale from 0 to 10]
   1. 0 (ignores concerns of others, dismisses, minimizes)
   2. 10 (validates concerns, restates them, summarizes them)
3. Rate the student’s ability to ask questions effectively: [Scale from 0 to 10]
   1. 0 (uses all closed ended questions)
   2. 10 (uses all open ended questions)
4. Rate the student’s ability to use appropriate body language: [Scale from 0 to 10]
   1. 0 (uses body language that shuts others out, crosses arms, enters others physical space)
   2. 10 (uses body statue, tone, and eye contact that encourages discussion)
5. Rate your emotional intelligence: [Scale from 0 to 10]
   1. 0 (emotions drive conversation, frustration, anger, lack of empathy)
   2. 10 (able to control emotions during encounter, uses empathy, understanding)]
6. Rate your overall approach to the conversation: [Scale from 0 to 10]
   1. 0 (made situation personal, needs to win)
   2. 10 (avoids making situation personal, remain patient-centered)
7. What are some ways you could improve your approach to this conversation? [*Free-text*]
