# Supplementary material for: Vital Conversations: An Interactive Conflict Resolution Training Session for Fourth-Year Medical Students
Source: MedEdPORTAL. 2021 Jan 25;17:11074. doi: 10.15766/mep_2374-8265.11074 (PMC7830754; doi:10.15766/mep_2374-8265.11074)
Supplement: Supplementary file 1 — Prework.docxTKI Teaching for Prework.docxVideo Realistic for Appendix A.mp4Video Empathic for Appendix A.mp4Rubric.docxClinical Encounter for Student.docxStandardized Patient Brief.docxPostwork.docxVideo 1 Conflict Resolution Postwork.mp4Video 2 Conflict Resolution Postwork.mp4 [file mep_2374-8265.11074-s001.zip › H. Postwork.docx]

## APPENDIX H

## Conflict Resolution Session: Postwork Student Evaluation

1. Please select your name from the following list. [*This question can be converted free-text.*]
2. Please select your coach and/or the faculty member who will be reviewing your video.

Please start the video of your clinical encounter. Answer the following questions based on your video. The standardized patient and your faculty mentor will provide you feedback based on this same rubric.

1. During this encounter, please note how long it took from the start of encounter until:

|  | Time (seconds) |
| --- | --- |
| The student to realize there was a conflict |  |
| The two individuals ask each other what their concerns/interests were |  |
| The two individuals to find a compromise |  |
| Total duration of the encounter |  |

1. Rate your ability to negotiate:

|  | Not at all | A little bit | A moderate amount | Mostly/Completely |
| --- | --- | --- | --- | --- |
| Identify/acknowledge there were different priorities/a problem |  |  |  |  |
| Break the problem down into smaller pieces |  |  |  |  |
| Acknowledge a shared goal |  |  |  |  |
| Summarize a compromise/commit to a plan that works for both parties |  |  |  |  |

1. Rate your ability to listen: [Scale from 0 to 10, selection]
   1. 0 (Ineffective listening, interrupts, explains too much)
   2. 10 (active listening, no interruptions)
2. Rate your ability to acknowledge the nurse’s concerns: [Scale from 0 to 10, selection]
   1. 0 (ignores concerns of others, dismisses, minimizes)
   2. 10 (validates concerns, restates them, summarizes them)
3. Rate your ability to ask questions effectively: [Scale from 0 to 10]
   1. 0 (uses all closed ended questions)
   2. 10 (uses all open ended questions)
4. Rate your ability to use appropriate body language: [Scale from 0 to 10]
   1. 0 (uses body language that shuts others out, crosses arms, enters others physical space)
   2. 10 (uses body statue, tone, and eye contact that encourages discussion)
5. Rate your emotional intelligence: [Scale from 0 to 10]
   1. 0 (emotions drive conversation, frustration, anger, lack of empathy)
   2. 10 (able to control emotions during encounter, uses empathy, understanding)]
6. Rate your overall approach to the conversation: [Scale from 0 to 10]
   1. 0 (made situation personal, needs to win)
   2. 10 (avoids making situation personal, remain patient-centered)
7. What are some ways you could improve your approach to this conversation? [*Free-text*]

We have asked two Capstone Faculty members to try to resolve the encounter with the standardized patient/nurse that you just had. Please watch the video, noting a few things that each faculty member did well and a few things that each could improve with conflict resolution.

**Video 1**: File “Conflict Resolution_PostWork_Video1” (Appendix I)

**Video 2**: File “Conflict Resolution_PostWork_Video2” (Appendix J)

1. What did each faculty member do well to resolve the conflict with the nurse? What could they improve one? How did their approach differ? [*Free-text*]
2. For the task of “participating as a contributing and integrated member of an interprofessional team” (EPA #9), how would you rate your entrustment based on what was presented to you in the brief?

Pre-entrustment (requires additional supervision before performing alone)

Entrustable (able to perform without any additional supervision)

1. How would you rate your own ability to work effectively as a member or leader of a healthcare team or other professional group? [*On a scale of 1 to 5, with half steps*]

Identifies benefits of interdisciplinary team-based care; Describes and appreciates the expertise of each team member, including the patient and family

[1]

Actively participates in team-based care and supports activities of other team members

[2]

Seeks leadership opportunities within professional organizations and is able to lead/facilitate meetings within the organization/system

[5]

Facilitates and leads team-based patient care activities; leads family/patient/ team conferences in an anticipatory manner and includes all relevant disciplines

[4]

Actively participates in interdisciplinary meetings to improve patient care; participates in family/patient/team member conferences and incorporates patient and family values and preferences

[3]

Not enough experience
