# Supplementary material for: Vital Conversations: An Interactive Conflict Resolution Training Session for Fourth-Year Medical Students
Source: MedEdPORTAL. 2021 Jan 25;17:11074. doi: 10.15766/mep_2374-8265.11074 (PMC7830754; doi:10.15766/mep_2374-8265.11074)
Supplement: Supplementary file 1 — Prework.docxTKI Teaching for Prework.docxVideo Realistic for Appendix A.mp4Video Empathic for Appendix A.mp4Rubric.docxClinical Encounter for Student.docxStandardized Patient Brief.docxPostwork.docxVideo 1 Conflict Resolution Postwork.mp4Video 2 Conflict Resolution Postwork.mp4 [file mep_2374-8265.11074-s001.zip › _Educational_Summary_Report_11074.pdf]

MedEdPORTAL® The AAMC Journal of Teaching and Learning Resources

Original Publication Open Access

Vital Conversations: An Interactive Conflict Resolution Training Session for Fourth-Year Medical Students

Rathnayaka Mudiyanselage Gunasingha, MD*, Nancy Knudsen, MD, Timothy Scialla, MD, Amanda Shepherd, MD, Alison Clay, MD

*Corresponding author: rg1430@outlook.com

Abstract

Introduction: The AAMC has recognized the importance of effective teamwork and collaboration. One core Entrustable Professional Activity emphasizes creating a climate of mutual respect and trust and prioritizing team needs over personal needs, which leads to safe, timely, effective, efficient, and equitable patient care. Relationship conflicts, specifically, are associated with decreased productivity, complex information processing, and work satisfaction. Given the prevalence of conflict and its impact on health care workers, the lack of conflict resolution curricula in undergraduate medical education is surprising. We developed a curriculum formally introducing these skills and allowing practice in a simulated environment before students entered residency. **Methods:** Fourth-year medical students completed a conflict resolution exercise in a mandatory transition-to-residency course. Students completed online prework including reflection on teamwork and information on conflict resolution styles, participated in a simulated conflict with a standardized patient acting as a nurse, and afterward completed a self-evaluation with video review by the students' assigned coach and feedback on the session. **Results:** We collected complete responses from 108 students. We evaluated the curriculum for feasibility and acceptability by faculty and students. Most students agreed with faculty on their entrustment and milestone levels. Students found that the session prompted self-reflection and was a good review of conflict resolution. The standardized patient and faculty feedback was found to be the most useful by the students. **Discussion:** We successfully implemented a simulated but realistic conflict resolution exercise. Students found the exercise helpful in their preparation for residency.

Keywords
Conflict Resolution, Curriculum, EPA, Communication Skills, Multimedia, Self-Assessment, Self-Regulated Learning, Standardized Patient

Educational Objectives

By the end of this session, learners will be able to:

1. Define the five different types of conflict resolution styles as described by the Thomas-Kilmann Conflict Instrument.
2. Recognize and reflect upon their own conflict resolution styles and learn how to utilize each style advantageously.
3. Critically evaluate a scenario where there is conflict between two people of similar or differing health care occupations.
4. Practice their own conflict resolution skills during a filmed standardized patient encounter.
5. Self-evaluate their own conflict resolution skills during a filmed standardized patient encounter.
6. Demonstrate skills for Entrustable Professional Activity 9: Interprofessional Teamwork.
7. Gain awareness of their behaviors during conflict from self-evaluation and feedback from the standardized patient and faculty.

Citation:
Gunasingha RM, Knudsen N, Scialla T, Shepherd A, Clay A. Vital conversations: an interactive conflict resolution training session for fourth-year medical students. *MedEdPORTAL*. 2021;17:11074.
https://doi.org/10.15766/mep_2374-8265.11074

Introduction

The AAMC has recognized important skills and tasks a graduating medical student should be able to do through its creation of core Entrustable Professional Activities (EPAs) for entering residency.[1] Core EPAs require direct observation and assessment in the workplace. In medical schools, these can come from faculty, mentors, residents, hospital staff, and/or peers.

Teamwork is included in EPA 9—"collaborate as a member of an interprofessional team"; this EPA includes skills that ensure safe, timely, effective, efficient, and equitable care of a patient.[1] It emphasizes creating a climate of mutual respect

Copyright © 2021 Gunasingha et al. This is an open-access publication distributed under the terms of the Creative Commons Attribution license.

and trust and prioritizing team needs over personal needs.[1] Conflicts, especially interpersonal ones, are associated with decreased productivity, complex information processing, and work satisfaction, as well as, in a health care environment, with patient safety issues.[2-9] However, observing and assessing skills within this EPA can be time and resource intensive. High-fidelity simulation and standardized patients are used to assess EPAs and correlate them to entrustment in clinical settings; they are also used as part of the process for medical board examinations.[10-12] Simulation allows for observation and assessment of skills in a time- and cost-effective environment.

While multiple undergraduate medical curricula have been developed for interprofessional communication, few address conflict management.[13-18] Those that do are discussion-based workshops or rely on discussion by an interprofessional team, which, while important, can be logistically difficult to schedule. Furthermore, these curricula do not allow individuals to practice skills in the heat of the moment in a conflict between two people.

We developed and implemented a unique curriculum for graduating medical students (1) to teach learners to critically analyze difficult communication styles for complex interprofessional encounters and (2) to allow them to practice conflict management skills in a simulated interprofessional conflict. By videotaping an encounter with a standardized patient acting as a nurse and using a novel rubric, learners and faculty were able to observe and assess specific behaviors in effective communication, and learners could self-reflect on how the situation could have been handled differently.

Methods

To formally teach and assess interprofessional skills prior to graduation, we implemented our new curriculum in a transition-to-residency course called Capstone. An annual longitudinal course, Capstone was designed to provide practical knowledge and skills to fourth-year medical students before they entered their intern year. In this mandatory course, all students were assigned to small groups consisting of eight to ten other students. Each group had a faculty member (the coach) who was assigned to students to provide individual feedback on actual patient encounters (e.g., writing a discharge summary) and simulated encounters (conducting informed consent or handoffs) and to conduct small-group sessions (high-fidelity simulation, difficult conversations, triage, medical ethics, etc.). The conflict resolution curriculum was created as a session with coaching within the Capstone course.

Faculty Preparation

All faculty coaches in Capstone (a paid position) were required to work in teams to design new curricula around the EPAs, including conflict resolution. The coaches also worked to develop agreement about entrustable and pre-entrustable behaviors for observed encounters as part of their faculty development. To train faculty for the conflict resolution session and to evaluate EPA 9, volunteer students participated in videotaped encounters with a standardized patient using a simulated conflict (see Resource Development, below, for further discussion of case development). For four pilot videos, the nurse role was filled by two different practicing nurses trained as standardized patients. The group reviewed the bulleted lists of pre-entrustable and entrustable behaviors from the AAMC.[19] Capstone faculty reviewed the pilot videos and defined specific types of behaviors that either escalated or de-escalated the conflict. After deciding on examples of behaviors, the group decided which behaviors represented pre-entrustment.[1,19] During these discussions, faculty also noted that program directors were often most worried about students who lacked insight into their own weaknesses; faculty decided that they would review students' self-assessments of their videotaped encounters. This would allow coaches to provide feedback to a student whose self-assessment was not accurate. That feedback was captured by adding a question asking if students' assessments were too harsh, were about right, or missed key points.

Resource Development

To prepare students for their standardized patient encounter, content was delivered virtually through prework (Appendices A-D). First, the students were introduced to different styles of conflict resolution and the situations in which various styles might be used to resolve conflict. Given its extensive use in the business field and its validated status, we also chose to introduce the Thomas-Kilmann Instrument (TKI).[20] The TKI conflict modes—accommodating, avoiding, collaborating, competing, compromising—would allow students to determine their primary style and to learn about the advantages and disadvantages of using each mode.[20-24] Demographic information was also collected as the teaching faculty wanted to use these data in iterative versions of the assignment (i.e., sharing prior classes' information about conflict resolution with future classes).

After students learned about different styles of conflict resolution, we wanted them to watch different conflicts and to consider what either escalated conflict or resolved conflict. For this activity, we wanted students to apply the same rubric that would be used to evaluate their standardized patient case. The Capstone team built

Copyright © 2021 Gunasingha et al. This is an open-access publication distributed under the terms of the Creative Commons Attribution license.

a rubric (Appendix E) for conflict based on two components of the standardized patient encounter: (1) general communication skills, such as body language, tone, and use of questions, utilized in our critical skills lab (CSL) for other activities and (2) the essential components of conflict resolution. The Capstone faculty reviewed the business literature on components of conflict resolution and considered the bulleted list of entrustable behaviors that were part of the core EPAs to identify any overlap.[1] Consistent overlap was found in four areas: identifying the problem, breaking the problem into pieces, agreeing on a common goal, and assigning responsibility. Faculty felt that students should watch a video and identify the time during the video when each aspect of conflict resolution was achieved. Likert scales used in our CSL for communication skills were used for other questions. The goal was to train students to critically evaluate behaviors in others prior to evaluating these behaviors in themselves following the standardized patient practical. We used Creative Commons videos depicting acceptable and unacceptable behavior. The videos were created by PhysicianHealthBC, a group that has made a number of videos about communication skills in medicine.[25,26]

Development of the Standardized Patient Case

Faculty working to develop content for the Capstone course met and discussed frequent conflicts they had observed between interns and nursing staff. Two situations were identified: (1) conflict around intravenous access and (2) conflict between teams about the best management for a patient needing dialysis. The faculty team developed both cases, including medical information about the patients, the team's anticipated plan, and a potential flashpoint for the nurse and the student that would spark conflict/different goals for each patient. The team piloted the cases with two students using practicing nurses as standardized patients. During this pilot, the development team discovered that the dialysis case was too medically complex and that students did not have the knowledge necessary for the case to develop.

After agreeing on the case that included disagreements about intravenous access between a nurse and the student, the CSL worked with the practicing nurses to develop it (see Appendices F and G). The practicing nurses provided ideas about behaviors that in real life would escalate a situation (e.g., not identifying the problem, not listening to the nurse, not recognizing how busy the nurse was, etc.) and which situations would help to resolve conflict (e.g., asking the nurse what ideas he or she had, how these types of situations had been handled in the past, etc.). During the pilot recordings, the Capstone course director, clinical skills director, and faculty who watched the videotaped pilots also felt it was helpful for the nurse to be distracted and busy in order to initiate conflict but also that the nurse should help the student succeed. The following year, two different nurses with standardized patient training (we had utilized them for other cases in our CSL lab) were hired for the case with a group of students who volunteered as trial participants. The nurses were trained with the videos from the initial pilot, along with feedback from the CSL and Capstone faculty. During the second pilot, approximately 40 students worked through the case; their videos were reviewed by Capstone faculty. At a debriefing meeting, faculty discussed how the case should be modified for future years; specifically, faculty felt that the nurse should offer the student some potential solutions if the student asked for help and that the nurse should be more willing to partner with the student and offer solutions that the student might not have considered simply due to lack of medical knowledge, such as changing some of the medication timing, stopping medications, or placing an external jugular line. In 2017, all Capstone students were required to complete the conflict resolution case.

Session Structure

The conflict resolution session consisted of three parts: prework, the standardized patient practical, and postwork. Each student was required to complete all three parts to receive credit for the session. The approach first emphasized the importance of conflict resolution, then allowed the learners to practice skills in a common scenario they would encounter in residency, and finally allowed them to reflect on their performance. Students also received feedback from the standardized patient immediately after the scenario and from their coach on their videotaped standardized patient encounter and their self-assessment.

Part 1: Prework

The prework (Appendices A-D) was designed to be a 1-hour exercise to teach learners about conflict resolution and critical analysis of a conflict in a medical setting. The prework was completed remotely and electronically using Qualtrics, an online data-collection tool our school used. A link was released 1-2 weeks before the session, and students were required to complete the assignment before the standardized patient practical.

Prework included information on conflict resolution modes, reflection on personal styles of conflict resolution, and video review of effective and ineffective conflict resolution in a medical context.[1,17] Appendix A introduces the two videos students were to view, along with Likert scales and free-text responses

Copyright © 2021 Gunasingha et al. This is an open-access publication distributed under the terms of the Creative Commons Attribution license.

used for evaluation of conflicts in medical settings. Appendix B consists of information on the TKI conflict resolution modes. Appendices C and D are the videos required for the prework exercise; any additional videos needed can be added by future curriculum designers.[25,26] Students were expected to look for specific behaviors in the provided videos that would facilitate effective conflict resolution; these were the same behaviors they would look for in their self-assessment of the practical portion.

Students were also provided with information about the conflict they would encounter with the standardized patient when they attended the practical. The encounter revolved around a single patient, the patient's primary nurse, and the need to obtain reliable vascular access. Specifically, students were asked to review the patient case (Appendix F) and to plan their potential conversation with the nurse. Students were provided with the rubric (Appendix E) as a handout that they, the standardized patient, and their coaches would use to review the encounter and to determine entrustment.

Part 2: Standardized Patient Practical
The Duke University School of Medicine's CSL had several rooms simulating patient care areas. These rooms incorporated cameras to videotape events for future review using Learning Space. The lab included standardized patient educators, trainers, and a director who oversaw these activities.

Middle-aged women served as our standardized patients, and each played the role of a nurse in an inpatient setting. The age and gender of the nurse (standardized patient) could be changed. Three standardized patients were hired and trained by the CSL utilizing prior videotapes from the pilot years with standardized patients who were nurses. Feedback from the faculty development group session before the start of Capstone 2017 was incorporated. Each standardized patient was hired with 4 hours of training. The standardized patient overview and brief are shown in Appendix G.

Students were required to sign up for the practical in an asynchronous manner. Signups included two other standardized patient encounters for the Capstone course (an informed consent session and a motivational interviewing session). A sample schedule is shown in Figure 1. The students heard a brief orientation and were allowed to review the encounter that had been previously provided to them. They then had a 20-minute session with a standardized patient that included the practical portion as well as immediate verbal feedback from the standardized patient.

	Conflict Negotiation		
	SP Name	Interview (10 min)	Verbal Feedback / Qualtrics (20 min) Plus Survey (40 min)
1		3:15-3:25	3:25-3:45 or 3:25-4:05
2		3:30-3:40	3:40-4:00 or 3:40-4:20
3		3:45-3:55	3:55-4:15 or 3:55-4:35
4		4:00-4:10	4:10-4:30 or 4:10-4:50
	Break	Break	Break
5		4:30-4:40	4:40-5:00 or 4:40-5:20
6		4:45-4:55	4:55-5:15 or 4:55-5:35
7		5:00-5:10	5:10-5:30 or 5:10-5:50
8		5:15-5:25	5:25-5:45 or 5:25-6:05

Figure 1. Sample student schedule. Abbreviation: SP, standardized patient.

Part 3: Self-Reflection and Evaluation
Immediately after the encounter, the standardized patient provided direct feedback to the student, based on the rubric provided in Appendix E. The rubric was similar to that used by students and faculty. Students then reviewed a video of their own performance during the standardized patient encounter and self-assessed using the rubric provided by the Capstone course and the video. Students were asked to evaluate components of their encounter and rate their entrustment as shown in Appendix H. Students were provided with the same list of entrustable behaviors that they had seen in the prework. The students' self-assessment and video were automatically sent to their Capstone coach using automated triggers within Qualtrics. Appendices I and J contain example conflict videos recorded by Duke's faculty for this course.

Each student's videoed standardized patient encounter was reviewed by the student's Capstone coach. The student received the coach's written feedback using automated email triggers available in Qualtrics. The faculty completed the evaluation of the encounter using the same rubric as the student. Faculty were also asked to assess whether the student's self-evaluation was accurate, was inaccurate and missed key findings, or was too harsh. All faculty met prior to the beginning of Capstone to review videos from a pilot session in order to agree on behaviors within those videos that represented entrustment and to provide feedback on how the student had played the case.

Time Commitment
Part 1, the prework, was self-directed and online. At most, a student needed 1 hour total to complete the exercise.

Parts 2 and 3 took place on the same day. The CSL team prepared student schedules, allowing time for students to review their videos prior to moving to the next station. Each session had one CSL staff member, whose involvement included orientation

Copyright © 2021 Gunasingha et al. This is an open-access publication distributed under the terms of the Creative Commons Attribution license.

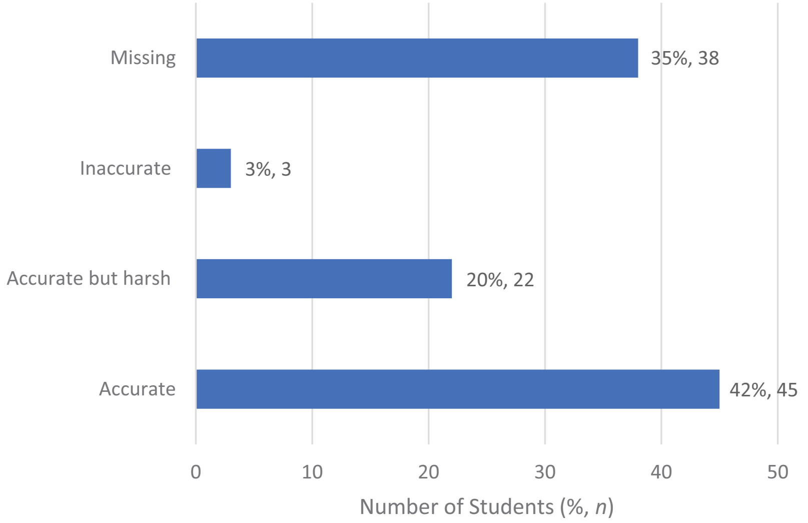

Figure 2. Accuracy of students' self-assessments of entrustment, as reviewed by faculty. Of note, there were 38 empty (missing) faculty responses.

to the exercise, timing of both the encounter and feedback sessions, manually starting and stopping video recordings of the students, and setting up the students for the postencounter video review and surveys.

Students had about 10 minutes to hear the pre-encounter orientation and complete the script review. They then had up to 10 minutes to undergo the encounter with the standardized patient. The standardized patient had 10 minutes at the end to give students verbal feedback. We had one staff member and two standardized patients for each day the practical was offered. After the encounter was over, the students had 40 minutes to watch their videotaped encounter and complete a Qualtrics survey.

Operational Factors

The school had a CSL with rooms simulating patient care areas and equipped to videotape sessions such as ours. We also had staff members trained in using this equipment. Each standardized patient was trained for about 4 hours on this specific clinical encounter. With one CSL staff member and two standardized patients, we were able to get 16 students through an encounter under 3 hours. For the CSL, the time required to have 100 students complete this exercise was 18-20 hours.

For students, the prework took about 30-45 minutes and the encounter with postwork took a total of 1 hour at most. The students' time commitment was a maximum of 2 hours.

Faculty were paid for their part as coaches in the entire Capstone curriculum. For this session, faculty were required to review the students' prework and video electronically and to provide written feedback. Time required for this was 30-45 minutes total.

Results

One hundred and ten fourth-year medical students took the required conflict resolution session during Capstone 2017. One hundred and eight students completed all parts of the session fully (prework, standardized patient encounter, and postwork). Thirteen faculty participated and completed evaluations.

Feasibility of a class-wide session was determined based on education outcomes and operational factors.

Education Outcome: Entrustment

After the standardized patient encounter, 94% of the students self-assessed themselves as entrustable, and 6% self-assessed themselves as pre-entrustable. Faculty assessed 91% of students as entrustable and 9% as pre-entrustable; this was not a statistically significant difference. Among those who were pre-entrustable, a common theme was the need to be more assertive. Overall, there was no difference in evaluation of entrustment as assessed by self or faculty.

It was optional for faculty to carefully review the students' self-assessment. The reason completion was optional was because faculty compensation had been determined prior to adding this question and the question required faculty to review not only the students' recorded sessions but also their prework and self-assessments (all sent at different times to the faculty's email). According to the faculty who reviewed students' videos and

Copyright © 2021 Gunasingha et al. This is an open-access publication distributed under the terms of the Creative Commons Attribution license.

self-assessments ($n = 70$), 96% of student assessments were accurate or accurate but harsh, and only 4% were inaccurate. Figure 2 shows how faculty reviewed the student assessments. For the completed 108 sets, we had both entrustment as judged by the student and entrustment as judged by faculty; of note, there were 38 empty responses for the specific question asking about the difference between student and faculty. While students and faculty were required to answer the question about how each would judge entrustment, faculty were not required to answer the question about difference, accounting for these empty responses.

Education Outcome: Value of Exercise to Students
The goal of the prework was to provide students with background in conflict resolution and to prompt reflection on behaviors during conflicts. Figure 3 shows the particular elements of the prework and how useful each was. The questions were rated on a Likert scale and were single choice. Eighty percent of students found learning the background of conflict resolution at least moderately useful, 76% found analyzing specific behaviors within videos at least moderately useful, and 65% found reviewing the videos of different conflicts at least moderately useful.

The students were also asked the value of the entire session. This question allowed for multiple choices. Figure 4 shows the summary of the students' choices. The session mainly served to prompt self-reflection. Of note, one-third of the class said that the session would change the way they would practice.

Finally, free-response feedback questions about the session were asked during the postwork portion. The answers below are organized by themes seen in the feedback.

- Most useful aspects of the curriculum that should be continued:
  - "Standardized patient encounter with feedback."
  - "Self-reflection on personal conflict resolution styles."
- Specific conflict resolution skills learned during the session that could be applied during residency:
  - "Active listening."
  - "Prioritize issues when resolving conflicts."
  - "Turn big problems into smaller, more manageable ones."
  - "Keep conversation patient-centered instead of provider-centered."
  - "Different conflict resolution styles can be used based on the particular scenario."
- Self-reflection on the session and behaviors learners noticed afterwards:
  - "Acute awareness of body language as a form of nonverbal communication."
  - "Allowed recognition of red flags in behavior during conflict for future awareness."
  - "Important to use techniques to set aside personal goals and listen."
- Changes or improvements to the session:
  - "Have standardized patient be more frustrated and harsh towards the learner for a tougher conflict."

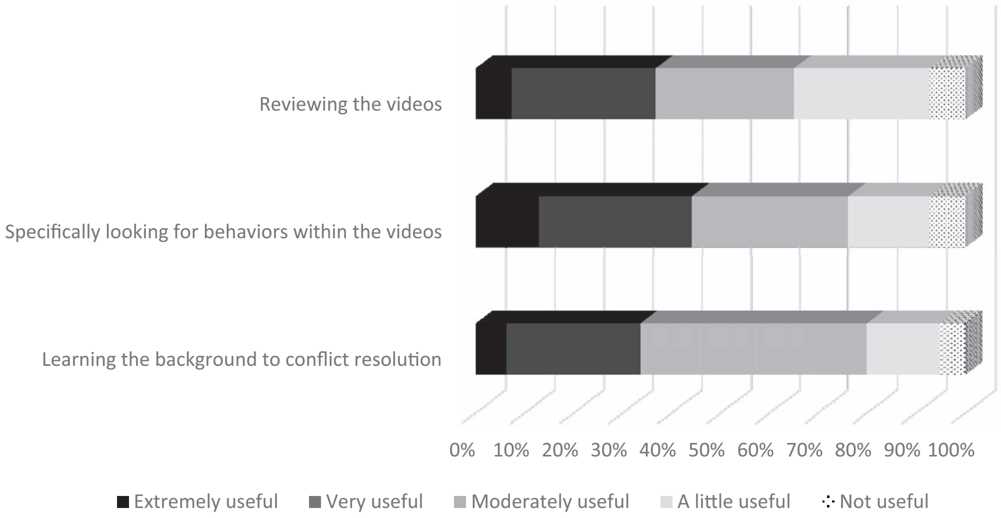

Figure 3. Value of prework elements for students.

Copyright © 2021 Gunasingha et al. This is an open-access publication distributed under the terms of the Creative Commons Attribution license.

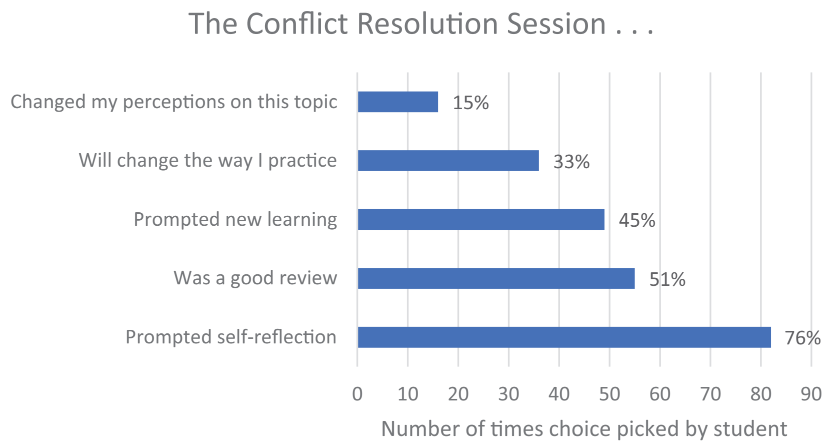

Figure 4. One hundred eight students were asked what was valuable about the session. The percentages represent the percentage of the class choosing each option.

- "Specific tips to on how to be more assertive while remaining polite and direct."
- "Have video review in small group setting with coach to get real time feedback."

Overall, students found the practical portion of the session with feedback to be the most useful aspect of the curriculum. There were certain aspects of their behavior they noted from self-review of their video and feedback that they could change for them to be more effective. For 76% of the students, the entire session prompted self-reflection of how they handle conflict.

Discussion

We were able to successfully implement a simulated but realistic conflict resolution training exercise. The session was designed to allow students to learn about, practice, and self-reflect on how they handled conflict. Heretofore, the practical sessions described in the literature had utilized group role-play and didactic discussion; this was the first time a standardized patient was used in a clinical scenario in conjunction with a rubric that allowed for dedicated practice of conflict resolution skills. Our school had the necessary infrastructure (the CSL) to implement the session described above. However, the session could be easily modified to accommodate schools that do not have the same capability. The important and required portions that make this a successful session are (1) an online lesson or survey system, (2) standardized patients, and (3) directed feedback from faculty.

We were able to demonstrate that this activity could assess entrustment and that students were able to reliably self-assess entrustment. This was most likely due to the students being trained to critically analyze conflict scenarios in the prework portion of the session and told that they were expected to apply similar behaviors in the practical portion. Although not explicitly indicated, the questions in the prework followed the rubric used by the student, standardized patient, and faculty for the student's evaluation. Students practiced recognizing effective and ineffective behaviors and were told the parameters of entrustment prior to their simulated scenario. Although many studies, including that of Kruger and Dunning, have shown that self-assessment can be inaccurate, others have demonstrated that people can gain insight from watching the behaviors of others and understanding why a failure has occurred.[27,28] Self-determined entrustment is possible when combined with reflection, skill building, receipt of feedback, and support from supervisors—all of which were included in this curriculum. This session was able to also identify a small portion of students in the pre-entrustment zone who might require extra practice before entering intern year. For these students, the best skill-builder would be additional standardized patient scenarios with feedback. As some learners suggested, watching the video in a small-group setting with faculty could serve as an additional recourse for those in the pre-entrustment zone.

Students found the session useful, especially for the feedback provided to them. Many did not realize that body language played a large part in how a message was conveyed, and most only became aware of their body language after the standardized patient and faculty evaluators gave them feedback. This type of feedback helped make the students more aware of habitual behaviors that they used in real conflicts. Students also found the session to promote self-reflection on their behaviors during conflict, which was key to improving weaknesses. Our questions in the postwork asked students to analyze particular aspects

Copyright © 2021 Gunasingha et al. This is an open-access publication distributed under the terms of the Creative Commons Attribution license.

of their behavior, which allowed them to dive deeper into the reasons the conflict ended the way it did.

We feel that it will be practical for most schools to replicate this curriculum and alter the scenario as needed for their standardized patients. In contrast to other published sessions, this particular session relies on student-driven prework and observation of an encounter, rather than on creating interprofessional teams to work through scenarios. Both approaches are valuable; however, this particular scenario does not require the logistics of creating interprofessional teams. Having coaches perform video review also overcomes the barrier of scheduling sessions when both faculty and students are available. Moving forward, the scenario could be adjusted to fit different environments and help students going into different fields practice conflict situations they could potentially encounter in the real world during intern year. Of note, students review the scenario before the practical to allow them to consider the medical knowledge aspects of the case. To build upon this curriculum, it would be interesting to have one scenario students are aware of beforehand (as this curriculum was designed) and another that is revealed only immediately prior to the encounter. The scenario that happens more organically (i.e., is unknown to the student until right before the standardized patient session) might also increase task-oriented stress since the student would have to figure out solutions in real time.

Our curriculum had some limitations. First, we had only one standardized patient scenario. To see growth and application of skills learned in practiced simulation, we could create additional scenarios. This would also be helpful to students, depending on the field they are going into. For example, students going into surgery would most likely deal with conflict in the operating room, and a simulated scenario in that setting would better resemble their experience. Second, our scenario took place between an intern and a nurse. We chose this particular conflict because it would be a common scenario for all interns across specialties. Third, the coaches in our session were paid for being a faculty member in the larger Capstone. Institutions with financial limitations may find this difficult to implement based on the number of students going through the session. An alternative method would be to have a smaller, dedicated group of faculty who review all the students going through the session instead of having one faculty member for every eight to 10 students. However, schools already utilizing CSL labs could make use of standardized patients for providing feedback, course/clerkship directors already financially supported could review videos as part of a clerkship, and/or institutions with fewer resources could utilize peer-to-peer teaching or even use residents who are in a residents-as-teachers track in their graduate medical education program. Finally, there was subjectivity in our rubric. Due to time and faculty constraints, we were not able to calculate interrater reliability amongst the faculty, especially for the determination of the amount of times a behavior was observed and for the Likert-scaled questions in the rubric. Ideally, as the faculty came to a group consensus on the appropriate behaviors, we should have had them complete the rubric for a series of students to make sure they were standardized graders.

Of note, for more advanced learners such as residents, the scenarios could be made more complicated and based on real-life events. This would allow those learners to have a sense of realism and also teach others in their position about addressing similar conflicts.

Overall, we feel that the session went well, was easy to run, and provided students with a realistic simulation of an encounter they will experience in residency. For the next iteration, we will provide two to three different scenarios, each with varying difficulty, with the option for students to do more than one for practice. Based on faculty availability, we would also like to include a session with old student videos and the rubric to make sure there is no interrater variability within the faculty prior to the start of the course. Given that fourth-year medical students go from being students to sudden leaders on clinical teams, more medical schools should give students practical experience in a controlled setting to recognize their strengths and weaknesses. Conflict could happen between peers (fellow interns or residents), medical students, supervising physicians, and administrative staff, to name just a few in the hospital environment. Each of these conflicts would be handled slightly differently given the social dynamics. Having scenarios with differing standardized patients (nurse vs. resident vs. supervising physician) would allow students to formulate different approaches to conflicts. Some students may be better in one scenario compared to another, and feedback will help them improve in the conflict resolution modes they do not use enough. Having a well-developed, interactive conflict resolution session would allow rising interns to practice dealing with situations that they will inevitably encounter in the wards and set them up to establish relationships with other health care professionals that enhance workflow, productivity, and patient safety.

Appendices

A. Prework.docx

B. TKI Teaching for Prework.docx

Copyright © 2021 Gunasingha et al. This is an open-access publication distributed under the terms of the Creative Commons Attribution license.

C. Video Realistic for Appendix A.mp4

D. Video Empathic for Appendix A.mp4

E. Rubric.docx

F. Clinical Encounter for Student.docx

G. Standardized Patient Brief.docx

H. Postwork.docx

I. Video 1 Conflict Resolution Postwork.mp4

J. Video 2 Conflict Resolution Postwork.mp4

All appendices are peer reviewed as integral parts of the Original Publication.

Rathnayaka Mudiyanselage Gunasingha, MD: Resident, Department of Surgery, Walter Reed National Military Medical Center; ORCID: https://orcid.org/0000-0002-1132-4559

Nancy Knudsen, MD: Professor, Department of Anesthesiology, and Associate Professor, Department of Surgery, Duke University School of Medicine

Timothy Scialla, MD: Adjunct Assistant Professor, Department of Medicine, Duke University School of Medicine

Amanda Shepherd, MD: Clinical Assistant Professor, Department of Medicine, University of Washington School of Medicine

Alison Clay, MD: Assistant Professor, Department of Surgery, and Assistant Professor, Department of Medicine, Duke University School of Medicine

Disclosures
None to report.

Funding/Support
None to report.

Ethical Approval
Reported as not applicable.

Disclaimer
The opinions or assertions contained herein are the private ones of the authors and are not to be construed as official or reflecting the views of the Department of Defense, the United States Army, Walter Reed National Military Medical Center, or any other agency of the US Government.

References

1. *Core Entrustable Professional Activities for Entering Residency: Curriculum Developers' Guide. Association of American Medical Colleges*; 2014.
2. Jehn KA, Bendersky C. Intragroup conflict in organizations: a contingency perspective on the conflict-outcome relationship. *Res Organ Behav*. 2003;25:187-242. http://doi.org/10.1016/S0191-3085(03)25005-X
3. Riskin A, Erez A, Foulk TA, et al. The impact of rudeness on medical team performance: a randomized trial. *Pediatrics*. 2015;136(3):487-495. http://doi.org/10.1542/peds.2015-1385
4. Poncet MC, Toullic P, Papazian L, et al. Burnout syndrome in critical care nursing staff. *Am J Respir Crit Care Med*. 2007;175(7):698-704. http://doi.org/10.1164/rccm.200606-806OC
5. Embriaco N, Azoulay E, Barrau K, et al. High level of burnout in intensivists: prevalence and associated factors. *Am J Respir Crit Care Med*. 2007;175(7):686-692. http://doi.org/10.1164/rccm.200608-1184OC
6. Wright KB. A communication competence approach to healthcare worker conflict, job stress, job burnout, and job satisfaction. *J Healthc Qual*. 2011;33(2):7-14. https://doi.org/10.1111/j.1945-1474.2010.00094.x
7. Winstanley S, Whittington R. Anxiety, burnout and coping styles in general hospital staff exposed to workplace aggression: a cyclical model of burnout and vulnerability to aggression. *Work Stress*. 2002;16(4):302-315. http://doi.org/10.1080/02678370210000058650
8. Görgens-Ekermans G, Brand T. Emotional intelligence as a moderator in the stress–burnout relationship: a questionnaire study on nurses. *J Clin Nurs*. 2012;21(15-16):2275-2285. http://doi.org/10.1111/j.1365-2702.2012.04171.x
9. Friedman RA, Tidd ST, Currall SC, Tsai JC. What goes around comes around: the impact of personal conflict style on work conflict and stress. *Int J Confl Manag*. 2000;11(1):32-55. https://doi.org/10.1108/eb022834
10. Dora-Laskey A, Sule H, Moadel T, et al. Entrustable Professional Activity 10: recognizing the acutely ill patient—a delirium simulated case for students in emergency medicine. *MedEdPORTAL*. 2016;12:10512. http://doi.org/10.15766/mep_2374-8265.10512
11. Royce CS, Atkins KM, Mendiola M, Ricciotti H. Teaching patient handoffs to medical students in obstetrics and gynecology: simulation curriculum and assessment tool. *MedEdPORTAL*. 2016;12:10479. http://doi.org/10.15766/mep_2374-8265.10479
12. Weersink K, Hall AK, Rich J, Szulewski A, Dagnone JD. Simulation versus real-world performance: a direct comparison of emergency medicine resident resuscitation entrustment scoring. *Adv Simul (Lond)*. 2019;4:9. http://doi.org/10.1186/s41077-019-0099-4
13. Goldberg C, Tanabe T, Plato M. Can we talk? Case studies in communication challenges and conflict management for clinicians. *MedEdPORTAL*. 2011;7:7957. http://doi.org/10.15766/mep_2374-8265.7957
14. Welch J, Jimenez H, Allen S. Teamwork & conflict management workshop. *MedEdPORTAL*. 2013;9:9605. http://doi.org/10.15766/mep_2374-8265.9605

Copyright © 2021 Gunasingha et al. This is an open-access publication distributed under the terms of the Creative Commons Attribution license.

15. Wellmon R, Knauss L, Gilin B, et al. Use of a video-based case study to promote interprofessional learning and collaboration skills. *MedEdPORTAL*. 2013;9:9612. http://doi.org/10.15766/mep_2374-8265.9612

16. Willgerodt M, Sonney J, Liner D, Barchet L. The power of a team: using unfolding video cases in interprofessional education for advanced health trainees. *MedEdPORTAL*. 2018;14:10707. http://doi.org/10.15766/mep_2374-8265.10707

17. Shrader S, Zaudke J. Crucial conversations and conflict resolution interprofessional simulation. *MedEdPORTAL*. 2015;11:10063. http://doi.org/10.15766/mep_2374-8265.10063

18. Wolfe AD, Hoang KB, Denniston SF. Teaching conflict resolution in medicine: lessons from business, diplomacy, and theatre. *MedEdPORTAL*. 2018;14:10672. http://doi.org/10.15766/mep_2374-8265.10672

19. Brown D, Gillespie C, Warren J. *Core Entrustable Professional Activities for Entering Residency—EPA 9 Schematic: Collaborate as a Member of an Interprofessional Team*. Association of American Medical Colleges; 2017.

20. Thomas KW, Kilmann RH. *An overview of the Thomas-Kilmann Conflict Mode Instrument (TKI)*. Kilmann Diagnostics. Accessed November 1, 2017. http://www.kilmanndiagnostics.com/overview-thomas-kilmann-conflict-mode-instrument-tki

21. Thomas KW, Kilmann RH, Bonnie B. Thomas-Kilmann conflict styles. In: *Project Leadership Training Manual. Family Voices of California*; 2001: chapter 4. https://www2.waisman.wisc.edu/cedd//WiFLI_S3/THOMAS_KILMANNConflictStyles.pdf

22. Thomas KW, Kilmann RH. *Thomas-Kilmann Conflict Mode Instrument*. Xicom; 1974.

23. Thomas KW, Kilmann RH. *Thomas-Kilmann Conflict Mode Instrument Profile and Interpretive Report*. CPP, Inc; 2008.

24. Thomas KW. Conflict and conflict management: reflections and update. *J Organ Behav*. 1992;13(3):265-274. http://doi.org/10.1002/job.4030130307

25. PhysicianHealthBC. *Resident is yelled at by a consultant - realistic*. YouTube. 2011. https://youtu.be/CyrSuY5g8iA

26. PhysicianHealthBC. *Resident is yelled at by a consultant - empathic*. YouTube. 2011. https://youtu.be/VHhqSIbY51s

27. Sagasser MH, Kramer AWM, Fluit CRMG, van Weel C, van der Vleuten CPM. Self-entrustment: how trainees' self-regulated learning supports participation in the workplace. *Adv Health Sci Educ Theory Pract*. 2017;22(4):931-949. http://doi.org/10.1007/s10459-016-9723-4

28. Kruger J, Dunning D. Unskilled and unaware of it: how difficulties in recognizing one's own incompetence lead to inflated self-assessments. *J Pers Soc Psychol*. 1999;77(6):1121-1134. https://doi.org/10.1037//0022-3514.77.6.1121

Received: February 1, 2020
Accepted: September 30, 2020
Published: January 25, 2021

Copyright © 2021 Gunasingha et al. This is an open-access publication distributed under the terms of the Creative Commons Attribution license.
